# Rapid assessment of communication consistency: sentiment analysis of public health briefings during the COVID-19 pandemic

**DOI:** 10.3934/publichealth.2022020

**Published:** 2022-02-10

**Authors:** Okan Bulut, Cheryl N. Poth

**Affiliations:** Centre for Research in Applied Measurement and Evaluation, Faculty of Education, University of Alberta, Edmonton, AB, Canada

**Keywords:** COVID-19, public health informatics, data mining, sentiment analysis, communication

## Abstract

**Background:**

A key component of the initial public health response to the COVID-19 pandemic involved the use of mass media briefings led by public health officials to communicate updates during a time of great uncertainty and rapidly changing information. This study aims to examine the consistency of communications expressed during the public health briefings to generate novel insights about the type, direction, and strength of public health messages. The data source included 131 readily accessible public health briefings alongside the provincial and national new confirmed case counts during the first two waves of rapidly increasing cases during the pandemic in Alberta, Canada. We employed sentiment analysis as a text mining technique to explore the types and frequency of words in public health briefings conveying positive and negative sentiments. Using statistical analyses and data visualizations, we examined how public health messaging shifted with case trends.

**Results:**

Our findings indicate consistent public health messaging in terms of sentiments regardless of case count fluctuations, an association of specific words with conveying positive and negative sentiments, and a focus on particular message patterns at different points during the first two waves of the COVID-19 pandemic.

**Conclusion:**

Our findings demonstrate the practical implications and methodological advantages of using sentiment analysis as a data analytics tool for rapidly and objectively assessing the consistency of health communications during a public health crisis.

## 1. Introduction

The public health response to COVID-19, an infectious disease caused by a novel coronavirus named severe acute respiratory syndrome coronavirus 2 (SARS-CoV-2), and the declaration of a pandemic by the World Health Organization on March 11, 2020, has greatly changed the way people live their lives around the world. The COVID-19 pandemic has been not only a significant public health threat for people around the world but also a major challenge for governments, public health authorities, and mass media as potential sources of health information [1],[2]. For the public, the COVID-19 pandemic has been marked by an increased quantity and massive flow of health information from multiple sources including traditional media (e.g., newspapers, magazines, and news channels on television and radio), social media platforms (e.g., Twitter, Instagram, and YouTube), and public health briefings of local, national, and international health authorities [3]. Since the beginning of the pandemic, public health experts warned repeatedly that early and consistent public messaging is highly important to convince people to act in ways that prevent COVID-19 transmission [4],[5]. Yet assessments of the extent to which and how these public health communications were consistent or changeable remains underreported.

Effective health communication during public health crises is very important because people seek credible sources of information to understand health risks and ways to cope with them [6]–[8]. A trustworthy source of COVID-19 information is required to promote COVID-19 prevention behaviors and to improve vaccine uptake [9]. According to the Centers for Disease Control and Prevention's (CDC) Crisis and Emergency Risk Communication (CERC) manual, the goals of health communications at the onset of and throughout a pandemic should be twofold: to inform the public about ongoing developments and to foster trust and credibility with the intended outcomes of convincing the public to follow public health recommendations during the pandemic [10]. What remains to be investigated is the extent to which these public health goals are achieved in practice and how the consistency of public health messaging contributes to a novel perspective. However, there are no well-established methods for measuring the consistency of public health messaging systematically. Using text mining tools, this study aims to investigate the consistency of communications expressed through the public health briefings to generate novel insights about the type, direction, and strength of public health messages.

### 1.1. Health communication practices during COVID-19

Effective and accurate health communication is a vital tool in the fight against health emergencies. Key documents from CDC [10], World Health Organization [11], and other health organizations provide guidance specific to good risk communication based on lessons learned from past pandemics and epidemics. For example, the CDC guidelines [10] offer salient guidance related to a template for effective messaging during an outbreak and describe characteristics that contribute to a messenger being perceived as trusted and credible. For the former, the CDC guidelines advise starting with empathy, explaining the threat, what is currently known, what actions are being taken and why, and concluding with a commitment to the situation. For the latter, the descriptions refer to general traits such as empathy, honesty, dedication, and competence. What is missing from the CDC guidelines is a reference to how these messages and traits might change over time during an extended health crisis such that we have experienced during the COVID-19 pandemic. In addition to the content of public health messages, another key element is the extent to which the type, direction, and strength of sentiments and emotions evoked by public health messages remain consistent [12]–[14].

In the absence of sufficient information about COVID-19, public health officials have had to use the existing communication principles and adapt their messaging strategies to the evolving understanding of the pandemic, including symptoms, social distancing, quarantine, and isolation rules, treatment options, and vaccines [2]. The mass media briefings held by public health officials from local, regional, and national health agencies have provided an important window into the evolving messages about COVID-19. During the onset of the pandemic, the public health messages focused on explaining where COVID-19 originated from and how the virus spread among individuals along with steps to prevent its transmission and its inequitable impacts on people's lives. As several COVID-19 vaccines became available to prevent people from getting infected, public health officials have begun to apply their experiences about effective health communications to manage information about the relevant risks and benefits of vaccines.

While public health officials have and continue to struggle to find persuasive and effective communication approaches throughout the pandemic, we must investigate and derive lessons learned that can be applied to both the ongoing COVID-19 pandemic and public health emergencies in the future. During the current COVID-19 pandemic, information overload, including false news, conspiracy theories, myths, and magical cures, continue to be a major challenge for both public health officials who aim to share updated and accurate information with the public and researchers who aim to analyze and improve the effectiveness of public health messages [15]. A recent study by Poth and colleagues [16] suggests that not only the content but also sentiments and emotions underlying the public health messages have the potential to influence public opinion and behavior during the COVID-19 pandemic. Therefore, a more holistic approach is needed to evaluate the effectiveness of public health messaging by analyzing large amounts of data (e.g., public health briefings, news, and social media reactions) based on different aspects of public health messages (e.g., content, sentiments, and accuracy).

### 1.2. Using sentiment analysis to evaluate health communication

Sentiment analysis, also referred to as opinion mining, enables researchers to extract and interpret emotions expressed towards a particular subject in a written text [17]. Using natural language processing (NLP) techniques, sentiment analysis can help researchers determine the direction (i.e., positive or negative), type, and strength of sentiments expressed in any form of text (e.g., documents, customer reviews, and social media posts). During the COVID-19 pandemic, researchers have extensively used sentiment analysis to analyze public reaction to news and updates on COVID-19. For example, Barkur et al. [18] used sentiment analysis to gauge the feelings of people living in India towards the lockdown using their posts on Twitter. In a similar study, de Las Heras-Pedrosa et al. [19] used posts on Twitter, YouTube, Instagram, and Internet forums to determine the impact of risk communication on the emotions and sentiments expressed by the social media users in Spain. So far, however, there has been little discussion about the sentiments and emotions in the announcements of public health officials [16]. Sentiment analysis of public health announcements can provide valuable insights into public health messaging as a starting point for building more effective communication strategies during a complex public health emergency.

### 1.3. Current study

In Alberta—a western Canadian province—the mass media briefings became the primary source of information for the public during the COVID-19 pandemic. The chief medical officer of health in Alberta served as the primary spokesperson in the province and informed Albertans about public health policies and practices [20]. In this study, we performed sentiment analysis to examine the consistency of sentiments in Alberta's public health briefings during the first and second waves of the COVID-19 pandemic. Specifically, we aim to address the following research questions: (1) To what extent did the public health briefings in Alberta vary in terms of length and sentiments during the first two waves of the COVID-19 pandemic? and (2) Were the length and sentiments of the public health briefings associated with the number of daily confirmed new cases? We hypothesized that if Alberta's chief medical officer maintained a balanced approach in risk communication, then both the length of public health briefings and the type and intensity of sentiments conveyed through public health briefings would remain similar over time and between the first two waves of the pandemic.

## 2. Methods

### 2.1. Data sources

Our analysis focuses on two four-month periods during the COVID-19 pandemic: Wave 1 as the period between the first media briefing by the Government of Alberta (March 5, 2020) and two weeks after the beginning of Stage 2 of Alberta's relaunch strategy (July 1, 2020) and Wave 2 as the period between the early onset of the second wave in Alberta (November 1, 2020) and the announcement of Alberta's ease of Step 2 restrictions (March 1, 2021). Our primary source of data was the official media briefings of the Government of Alberta. Dr. Deena Hinshaw, who is the chief medical officer of health for the province of Alberta, has regularly participated in the media briefings and informed the public on the progress of the COVID-19 pandemic. Transcripts of the media briefings during Wave 1 (n = 72) and Wave 2 (n = 59) have been shared through the Government of Alberta's official website for the COVID-19 pandemic [21]. To conduct sentiment analysis on the media briefings, the transcript for each media briefing was downloaded from the Government of Alberta's official website and stored as a separate text document (i.e., a Microsoft Word document). The secondary data source was the provincial COVID-19 statistics for the same periods, including confirmed cases and their demographic information. The daily statistics and other pandemic-related information have been shared by the Government of Alberta via its interactive COVID-19 data app [22]. Statistical data covering the target timeframe of this study were extracted via the interactive data app as a single comma-separated values (CSV) file. The file included the date of reporting, Alberta Health Services Zone, gender, age group, case status (i.e., recovered, died, or active), and case type (i.e., probable or confirmed) for each patient. This patient-level data was aggregated by date to calculate the total number of daily confirmed cases. No ethical approval was required due to using publicly available data.

### 2.2. Data preparation

Programming scripts were written and implemented using the R programming language [23] to import, merge, and transform the textual data from the transcripts of the media briefings into a tidy data format that could be used for sentiment analysis. Several steps were performed to process and prepare the data. First, the sentences from the media briefings were converted into lowercase using the *stringr* package [24]. Second, punctuation and nonalphanumeric characters were removed from the textual data. Third, word tokenization was performed to split each sentence into individual words (i.e., tokens) using the *tidytext* [25] and *tokenizers* [26] packages. Fourth, lemmatization was applied to the tokens to resolve each word into its canonical form using the *textstem* package [27]. Lastly, stopwords (e.g., a, an, the, in) that frequently occur in the data but might not add much value to the meaning of the document were removed from the data. The final data set was merged with the data set involving the COVID-19 case statistics, using the date as a matching criterion.

### 2.3. Sentiment analysis

We used sentiment analysis to explore the types and frequency of words conveying positive and negative sentiments to generate novel insights about how public health messaging shifted with case trends. Specifically, we analyzed sentiments expressed by Alberta's chief medical officer of health through the official media briefings during the first two waves of the COVID-19 pandemic in Alberta, Canada. We performed two types of sentiment analysis using the media briefings. First, we used the BING lexicon [28] to categorize each word as either positive or negative, calculated the frequency of positive and negative words, and subtracted the number of negative words from the number of positive words to calculate a sentiment score for each media briefing. For example, the public health briefing for March 12, 2020, consisted of 37 positive words and 53 negative words based on the BING lexicon. Using these values, the sentiment score was calculated as 37 − 53 = −16, suggesting that the overall sentiment for the public health briefing of March 12, 2020, was negative. In this study, the sentiment scores ranged from −48 to 19 for the first two waves of the COVID-19 pandemic in Alberta. Second, we used the NRC Emotion Lexicon [29] to categorize each word based on Plutchik's [30] psych evolutionary theory of basic emotions (anger, fear, anticipation, trust, surprise, sadness, joy, and disgust) and two sentiments (positive and negative).

### 2.4. Statistical analysis

To address the research questions of this study, we analyzed the data using both descriptive and inferential statistics. First, we used descriptive statistics (i.e., mean, standard deviation, minimum, and maximum values of sentiment scores) and visualizations to analyze the variation in the length and sentiments of the public health briefings during the first two waves of the COVID-19 pandemic. Second, we performed a multiple regression analysis to examine whether the length and sentiments of the public health briefings (dependent variables) significantly varied based on the number of confirmed COVID-19 cases, waves of the pandemic, and their interactions (independent variables). The regression analysis was conducted using the R programming language [23].

## 3. Results

### 3.1. Variation in the length and sentiments of public health briefings

Table 1 shows a descriptive summary of the length and sentiment scores of the public health briefings during the first two waves of the COVID-19 pandemic. The results indicate that the length of the public health briefings seems to vary greatly (i.e., number of words) but not in sentiment scores. The third month of the first wave (i.e., May 2020) involved the lengthiest briefings while the third month of the second wave (i.e., January 2021) involved the least lengthy briefings across the eight-month period. The average sentiment scores for the entire period were negative, indicating that the public health briefings mostly conveyed a negative sentiment. The sentiment scores from the first wave seem to be slightly lower than those from the second wave.

**Table 1.** A descriptive summary of the length and sentiments of the public health briefings.

Wave	Month	Number of Words				Sentiment Score			
		*M*	*SD*	*Min*	*Max*	*M*	*SD*	*Min*	*Max*
1	March 2020 (*n* = 22)	361	93.9	137	594	−14	12.5	−48	5
1	April 2020 (*n* = 22)	378	114	133	591	−16.7	11.8	−37	13
1	May 2020 (*n* = 16)	450	131	210	730	−10.6	17.3	−47	16
1	June 2020 (*n* = 12)	380	55.4	301	507	−3	13.7	−28	17
2	November 2020 (*n* = 14)	439	174	104	747	−16.5	16.1	−45	5
2	December 2020 (*n* = 17)	309	72	166	414	−9.94	6.96	−24	−1
2	January 2021 (*n* = 15)	241	60.6	156	359	−8.87	4.84	−15	2
2	February 2021 (*n* = 13)	374	111	107	503	−9.38	12.7	−28	19

**Figure 1.**
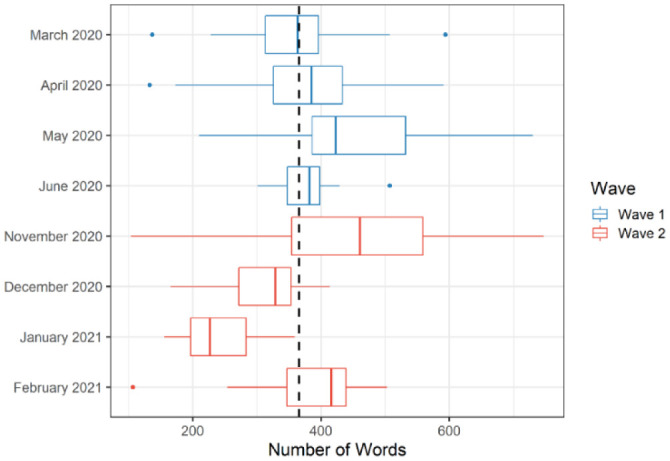
The number of words used in the public health briefings across the two waves of the COVID-19 pandemic in Alberta (*Note*: The dashed line represents the average number of words).

**Figure 2.**
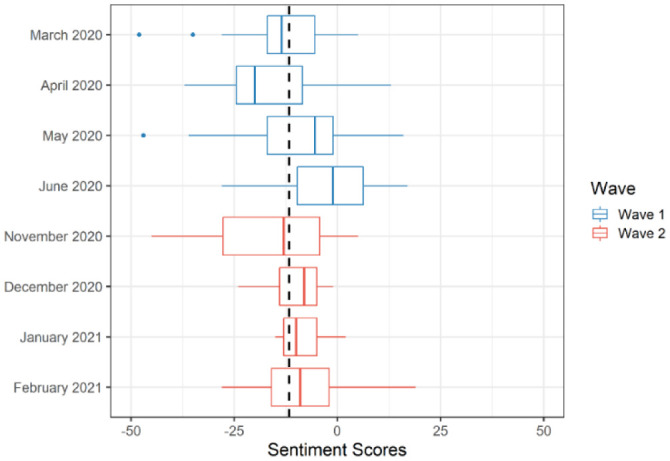
Sentiment scores for the public health briefings across the two waves of the COVID-19 pandemic in Alberta (*Note*: The dashed line in the figure represents the average sentiment score).

Figures 1 and 2 demonstrate the distributions of the length and sentiment scores of the public health briefings during the first two waves of the pandemic, respectively. In the figures, the dashed line represents the average length or sentiment scores (respectively) across the eight-month period. Waves 1 and 2 appear to have a reversed pattern in terms of the change in the length of the public health briefings (i.e., increase in the first three months and decrease in the fourth month, or the opposite). Furthermore, compared with the onset of the first wave (March 2020), the onset of the second wave (November 2020) seems to have a larger variation in terms of the number of words. A similar pattern was observed in terms of the sentiment scores (see Figure 2). Compared with March 2020, November 2020 had a lower sentiment score on average and varied slightly more. However, the distribution of the sentiment scores does not seem to fluctuate significantly across the eight months. Figure 2 also shows that the end of both waves (i.e., June 2020 and February 2021) involved more positive sentiments.

**Figure 3.**
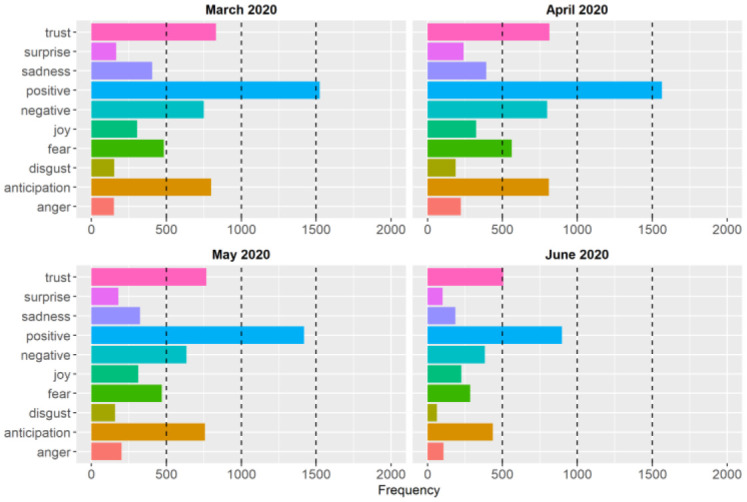
Frequencies of the basic emotions and sentiments expressed in the public health briefings during the first wave of the COVID-19 pandemic in Alberta.

**Figure 4.**
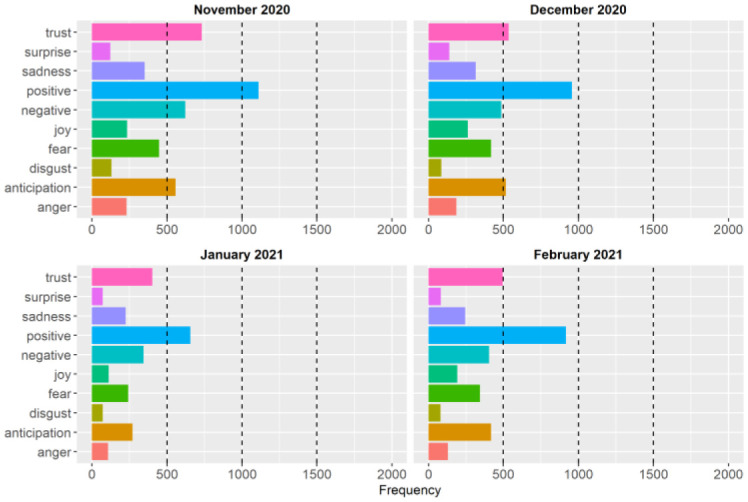
Frequencies of the basic emotions and sentiments expressed in the public health briefings during the second wave of the COVID-19 pandemic in Alberta.

In addition to the sentiment scores based on the difference between negative and positive sentiments, we also examined the type of basic emotions and sentiments expressed in the public health briefings. Figures 3 and 4 show the frequencies of the basic emotions and sentiments during the first and second waves of the pandemic, respectively. The figures show that positive and negative sentiments, trust, and anticipation were the most common emotions based on the words that Dr. Hinshaw delivered in her briefings during the first two waves. Although the overall pattern is very similar between the two waves, the frequencies of the sentiments and emotions are lower in the second wave because there were fewer public health briefings during the second wave (see Table 1).

### 3.2. Association between daily confirmed new cases and public health briefings

**Figure 5.**
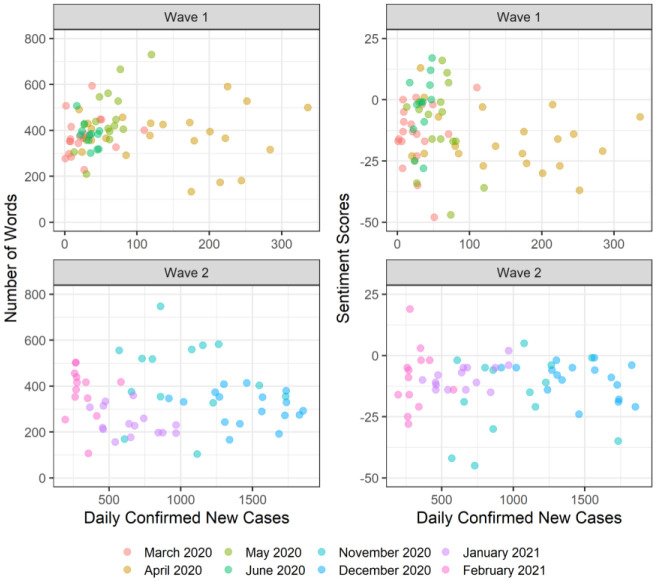
Scatterplots of daily confirmed new cases, the number of words, and sentiment scores of the public health briefings during the first two waves of the pandemic.

To address the associations among length, sentiments, and new cases, we performed multiple linear regression using the number of daily confirmed new cases in Alberta, waves (i.e., Wave 1 or Wave 2), and their interactions to predict the length and sentiment scores of the public health briefings. Table 2 shows the results from the two multiple regression models. The results indicated that the regression models did not explain a significant portion of the variance in the length of public health briefings (*F*(3, 125) = 2.60, *p* = 0.055, adjusted R^2^ = 0.04) and sentiment scores of public health briefings (*F*(3, 125) = 1.36, *p* = 0.257, adjusted R^2^ = 0.01). The number of daily confirmed new cases, waves, and their interactions were not statistically significant predictors. These findings suggest that Dr. Hinshaw's public health briefings remained consistent across the two waves of the pandemic, regardless of the time (i.e., waves) and severity of the pandemic outcomes (i.e., daily confirmed new cases). As a visual form of the regression results, Figure 5 also demonstrates the lack of an association between daily confirmed new cases, sentiment scores, and the length of public health briefings across the two waves.

**Table 2.** Results of the multiple regression models.

Predictors	Length (Number of Words)				Sentiment Score			
	*b*	*SE*	*t*	*p*	*b*	*SE*	*t*	*p*
Intercept	390.17	19.75	19.75	<0.001	−9.21	2.14	−4.31	<0.001
Daily new confirmed cases	0.05	0.19	0.26	0.796	−0.04	0.02	−1.96	0.052
Wave (Wave 1 is the reference)	−37.77	37.46	−1.01	0.315	−1.27	4.06	−0.31	0.755
Daily new confirmed cases × Wave	−0.07	0.19	−0.35	0.730	0.04	0.02	1.90	0.060

## 4. Discussion

Our study points to the consistency of public health messaging in Alberta, regardless of case counts and waves of the COVID-19 pandemic, evidenced by consistency in the strength, direction, and type of sentiments expressed in the public health briefings. The lack of correlation among the number of daily confirmed new cases, waves, and the sentiment scores is interesting to note because it provides evidence of consistency in public health messaging even when there are few cases. These findings provide essential insights and offer feasible procedures for health communication practice because risk communication guidelines often link consistency with trust and credibility of the public health messages [10],[11],[31],[32]. Previous research [2],[33] indicated that the lack of clear and consistent public health messaging pandemic could lead to unintended consequences such as negative reactions from the public against public health measures (e.g., school closures and vaccine acceptance). Furthermore, consistent messages from public health authorities are essential for promoting compliance with the public health measures [34]. Thus, guidance for effective public health messaging needs to be explicit about the role of stability and more attention should be paid to sentiments and emotions in the evaluation of public health messaging. This points to the need for new tools that can enable public health officials and researchers to rapidly assess the effectiveness of public health messaging in light of unfolding trends during a public health emergency. To date, sentiment analysis has been mostly used for evaluating the public reaction to health measures taken against the COVID-19 pandemic on social media platforms [35],[36]. However, the findings of our study indicated that scores obtained from sentiment analysis can also be used as a new metric to promote a more holistic evaluation of public health messaging.

Our study also provides unique insights from readily accessible datasets beyond a content analysis of public health messages during the COVID-19 pandemic. Specifically, sentiment analysis contributes to a timely understanding of what effective public health messaging looks like in practice. In so doing, sentiment analysis offers a real-time, data-driven tool for evaluating the consistency of public health messaging that is more useful than typical retrospective studies. This allows frontline public health communicators as well as those working behind the scenes to monitor and adjust the consistency of messages in light of a thorough understanding of type, direction, and strength of sentiments. Tools that are feasible to conduct rapid assessments of consistency of public messaging during evolving global outbreaks such as COVID-19 can account for influences on and from rapidly changing local, national, and international contexts and develop more holistic understandings of shifts in messaging in the face of uncertainty and changing information. Since the emergence of COVID-19 as a global pandemic, mass media communications have been used by local, national, and international public health agencies. Therefore, rapid assessments of consistency and change across message content and sentiments provide critical information to promote public health communication goals, particularly in the context of global pandemics.

## 5. Conclusions

The present study demonstrated a novel method (i.e., sentiment analysis) to review and evaluate the consistency of public health messaging during the COVID-19 pandemic. The results of our study indicate that sentiment analysis can be an effective data analytics tool for evaluating and improving the consistency of public health communication during public health crises. Local, national, and international health authorities can use sentiment analysis available in software programs such as RapidMiner to analyze the content of either written or verbal media briefings before sharing the content with the public. In this study, we demonstrated a lexicon-based sentiment analysis where we used the BING lexicon to categorize English words as positive and negative. However, sentiment analysis is not necessarily limited to the English language. Many lexicons are also available in other languages. For example, the Data Science Lab at Stony Brook University has a repository of open-source, multilingual sentiment lexicons in more than 80 major languages [37]. These sentiment lexicons can be used to evaluate the consistency of public health communication across multiple languages.

This study is the first step to understanding the unique contribution of public health official-led media briefings during the COVID-19 global pandemic. However, the study has certain limitations that are important to consider. First, our data collection was limited to one Canadian province over a prescribed period. Hence, results may not be generalizable to other contexts. Future studies should assess a broader scope of contexts and look at similarities and differences in the consistency of mass media public health communications. Second, this exploratory study utilized sentiment analysis to demonstrate the type, direction, and strength of sentiments in public health messages. Future studies should integrate additional data sources to explain the trends in public health messaging. Third, the actual impact of the messages on public behavior during the pandemic is still unknown. Members of the public may receive and process messages differently depending on their interpretation of the sentiment being conveyed. Future studies could seek the perspectives of the public health officials conveying the mass media briefings and members of the public to assess the extent of the impact on behavior.

## References

[1] Ratzan SC, Gostin LO, Meshkati N (2020). COVID-19: An urgent call for coordinated, trusted sources to tell everyone what they need to know and do. J Health Commun.

[2] Ratzan SC, Sommariva S, Rauh L (2020). Enhancing global health communication during a crisis: lessons from the COVID-19 pandemic. Public Health Res Pract.

[3] Austin EW, Austin BW, Willoughby JF (2021). How media literacy and science media literacy predicted the adoption of protective behaviors amidst the COVID-19 pandemic. J Health Commun.

[4] Benham JL, Lang R, Burns KK (2021). Attitudes, current behaviours and barriers to public health measures that reduce COVID-19 transmission: A qualitative study to inform public health messaging. Plos One.

[5] Yamanis T (2020). Clear, consistent health messaging critical to stemming epidemics and limiting coronavirus deaths.

[6] Austin L, Fisher LB, Jin Y (2012). How audiences seek out crisis information: exploring the social-mediated crisis communication model. J Appl Commun Res.

[7] Maitlis S, Sonenshein S (2010). Sensemaking in crisis and change: inspiration and insights from Weick (1988). J Manage Stud.

[8] Reynolds B, Seeger MW (2005). Crisis and emergency risk communication as an integrative model. J Health Commun.

[9] Latkin CA, Dayton L, Strickland JC (2020). An assessment of the rapid decline of trust in US sources of public information about COVID-19. J Health Commun.

[10] Centers for Disease Control and Prevention (2018). Crisis and emergency risk communication (CERC) manual.

[11] World Health Organization (2017). Communicating risk in public health emergencies: A WHO guideline for emergency risk communication (ERC) policy and practice.

[12] Agrawal N, Menon G, Aaker JL (2007). Getting emotional about health. J Mark Res.

[13] Aslam F, Awan TM, Syed JH (2020). Sentiments and emotions evoked by news headlines of coronavirus disease (COVID-19) outbreak. Hum Soc Sci Commun.

[14] Kwan JSL, Lim KH (2020). Understanding public sentiments, opinions and topics about COVID-19 using Twitter. 2020 IEEE/ACM International Conference on Advances in Social Networks Analysis and Mining (ASONAM).

[15] Sasidharan S, Singh DH, Vijay S (2020). COVID-19: Pan(info)demic. Turk J Anaesthesiol.

[16] Poth CN, Bulut O, Aquilina AM (2021). Using data mining for rapid complex case study descriptions: example of public health briefings during the onset of the COVID-19 pandemic. J Mix Method Res.

[17] Yi J, Nasukawa T, Bunescu R (2003). Sentiment analyzer: Extracting sentiments about a given topic using natural language processing techniques. Third IEEE international conference on data mining.

[18] Barkur G, Vibha GB (2020). Sentiment analysis of nationwide lockdown due to COVID 19 outbreak: Evidence from India. Asian Journal of Psychiatry.

[19] de Las Heras-Pedrosa C, Sánchez-Núñez P, Peláez JI (2020). Sentiment analysis and emotion understanding during the COVID-19 pandemic in Spain and its impact on digital ecosystems. Int J Environ Res Public Health.

[20] Government of Alberta (2020). Office of the Chief Medical Officer of Health.

[21] Government of Alberta (2020). COVID-19 info for Albertans.

[22] Government of Alberta (2020). COVID-19 Alberta statistics.

[23] R Core Team (2021). R: A language and environment for statistical computing.

[24] Wickham H (2019). stringr: Simple, consistent wrappers for common string operations.

[25] Silge J, Robinson D (2016). *tidytext*: Text mining and analysis using tidy data principles in R. JOSS.

[26] Mullen AL, Benoit K, Keyes O (2018). Fast, consistent tokenization of natural language text. JOSS.

[27] Rinker TW (2018). textstem: Tools for stemming and lemmatizing text.

[28] Hu M, Liu B (2004). Mining and summarizing customer reviews. Proceedings of the ACM SIGKDD International Conference on Knowledge Discovery & Data Mining (KDD-2004).

[29] Mohammad SM, Turney PD (2013). Crowdsourcing a word–emotion association lexicon. Comput Intell-US.

[30] Plutchik R, Plutchik R, Kellerman H (1980). A general psychoevolutionary theory of emotion. Emotion: Theory, research and experience, Theories of emotion.

[31] Savoia E, Lin L, Viswanath K (2013). Communications in public health emergency preparedness: a systematic review of the literature. Biosecur Bioterror.

[32] Tumpey AJ, Daigle D, Nowak G, Rasmussen SA, Goodman RA (2018). Communicating during an outbreak or public health investigation. The CDC field epidemiology manual.

[33] Leask J, Hooker C (2020). How risk communication could have reduced controversy about school closures in Australia during the COVID-19 pandemic. Public Health Res Pr.

[34] Hung L, Lin M (2022). Clear, consistent and credible messages are needed for promoting compliance with COVID-19 public health measures. Evid Based Nurs.

[35] Wang T, Lu K, Chow KP (2020). COVID-19 sensing: negative sentiment analysis on social media in China via BERT model. IEEE Access.

[36] Aljameel SS, Alabbad DA, Alzahrani NA (2021). A sentiment analysis approach to predict an individual's awareness of the precautionary procedures to prevent COVID-19 outbreaks in Saudi Arabia. Int J Environ Res Public Health.

[37] Chen Y, Skiena S (2014). Building sentiment lexicons for all major languages. Proceedings of the 52nd Annual Meeting of the Association for Computational Linguistics.

